# Supplementary figures and images for: Structural Analysis of the A Mating Type Locus and Development of the Mating Type Marker of Agaricus bisporus var. bisporus
Source: J Fungi (Basel). 2023 Feb 21;9(3):284. doi: 10.3390/jof9030284 (PMC10051438; doi:10.3390/jof9030284)

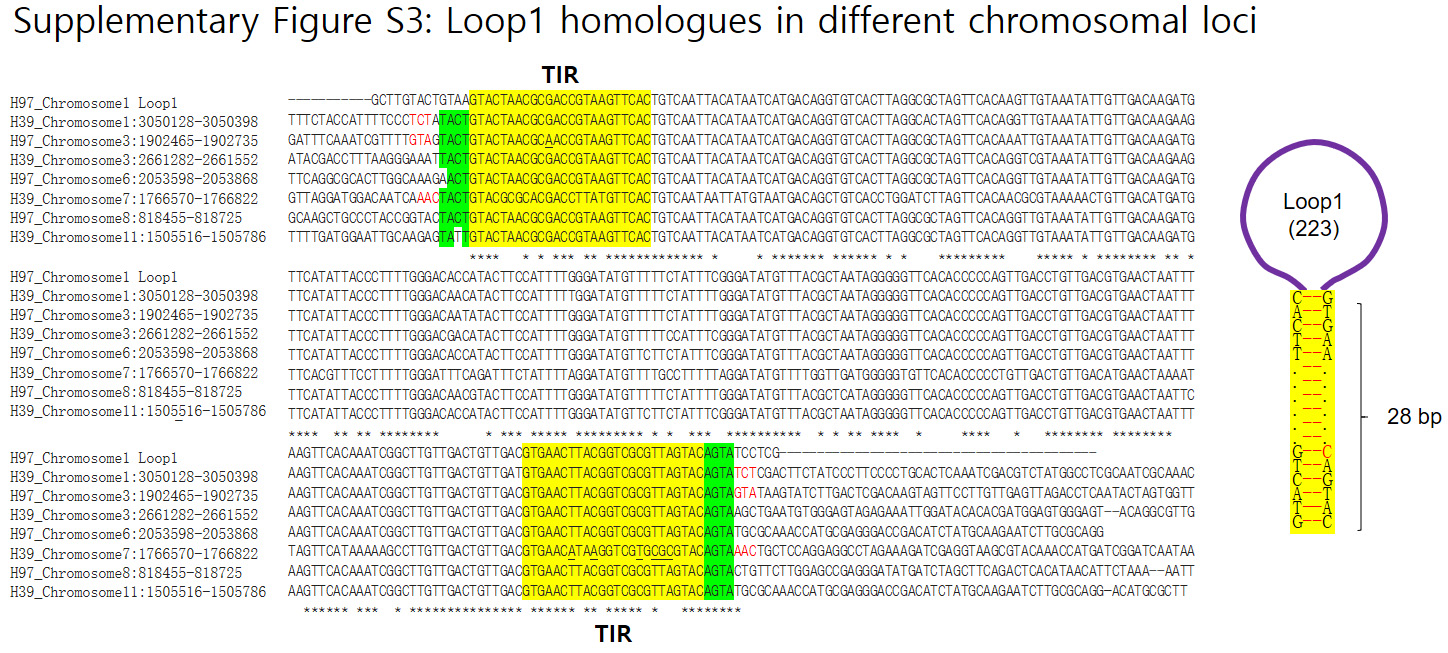

Supplement: Supplementary file 1 [file jof-09-00284-s001.zip › Supplementary Figure S3.TIF]

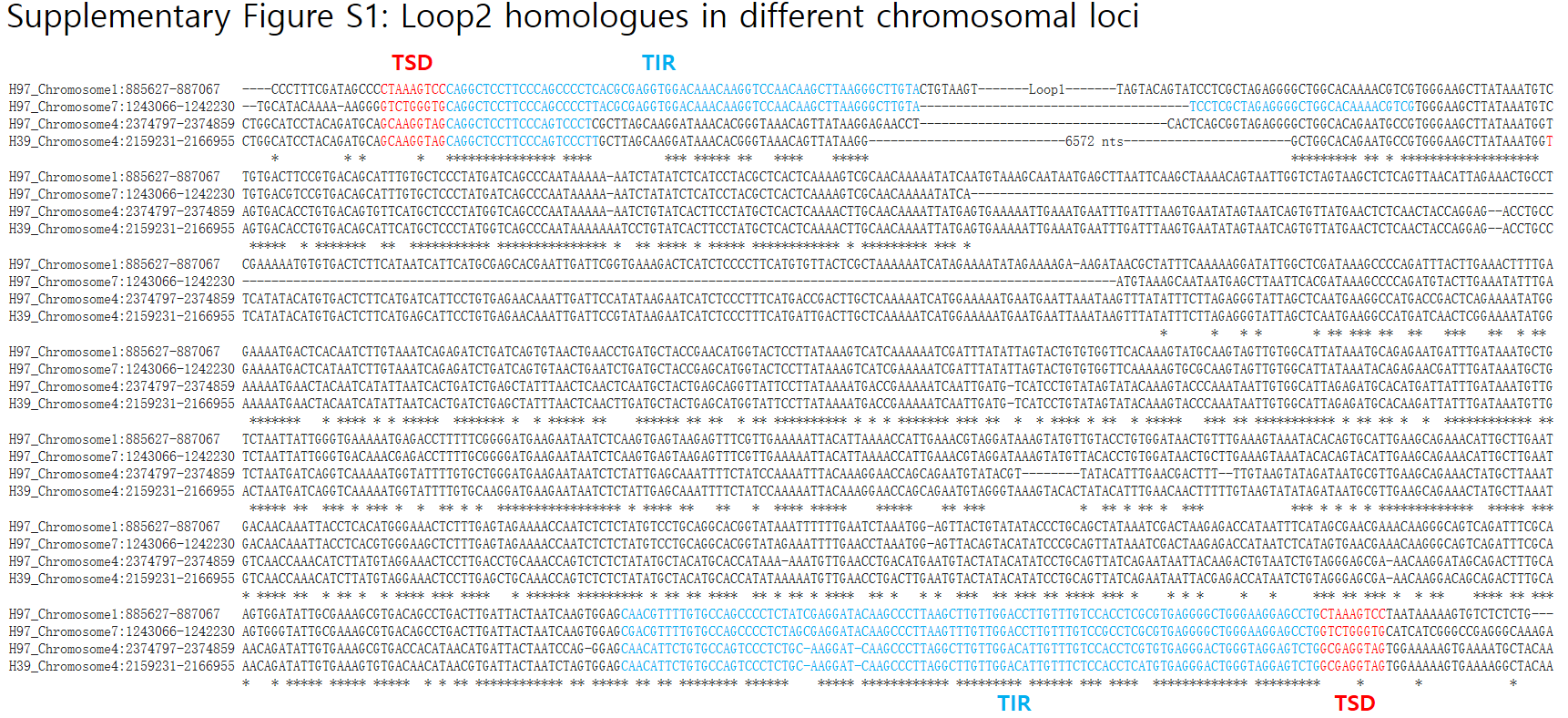

Supplement: Supplementary file 1 [file jof-09-00284-s001.zip › Supplementary Figure S1.TIF]

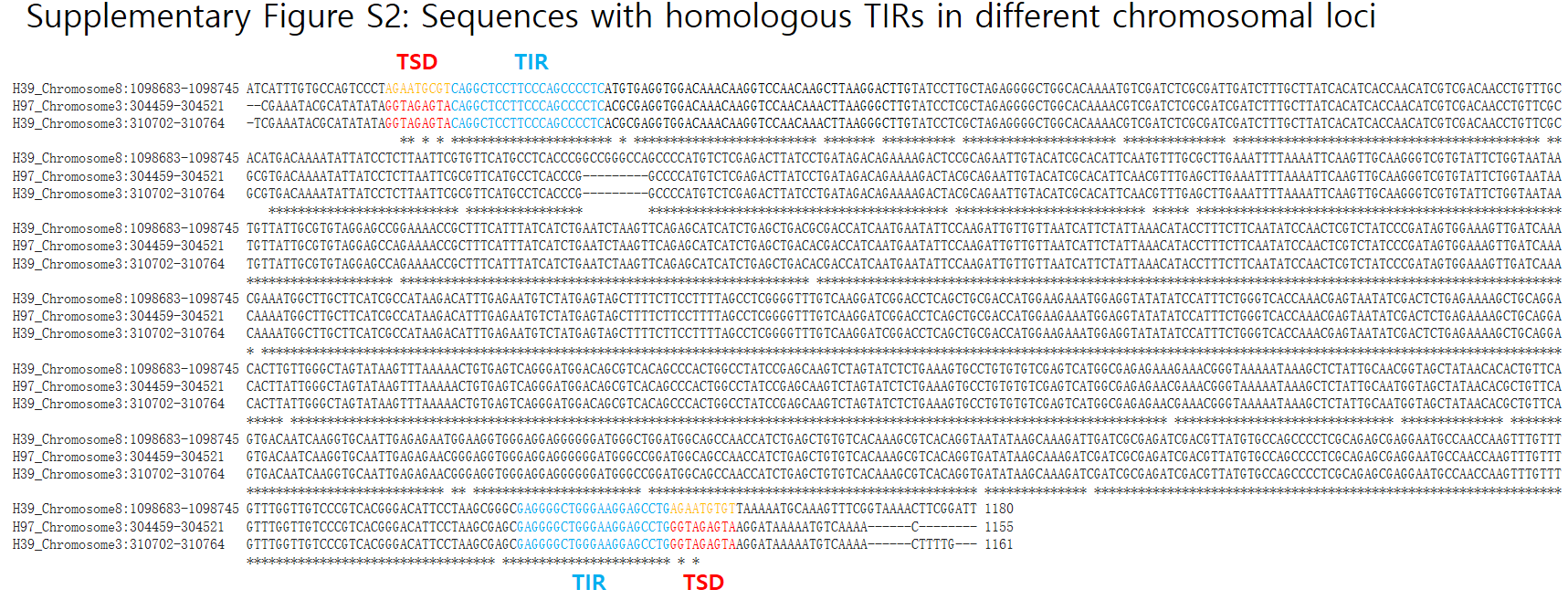

Supplement: Supplementary file 1 [file jof-09-00284-s001.zip › Supplementary Figure S2.TIF]

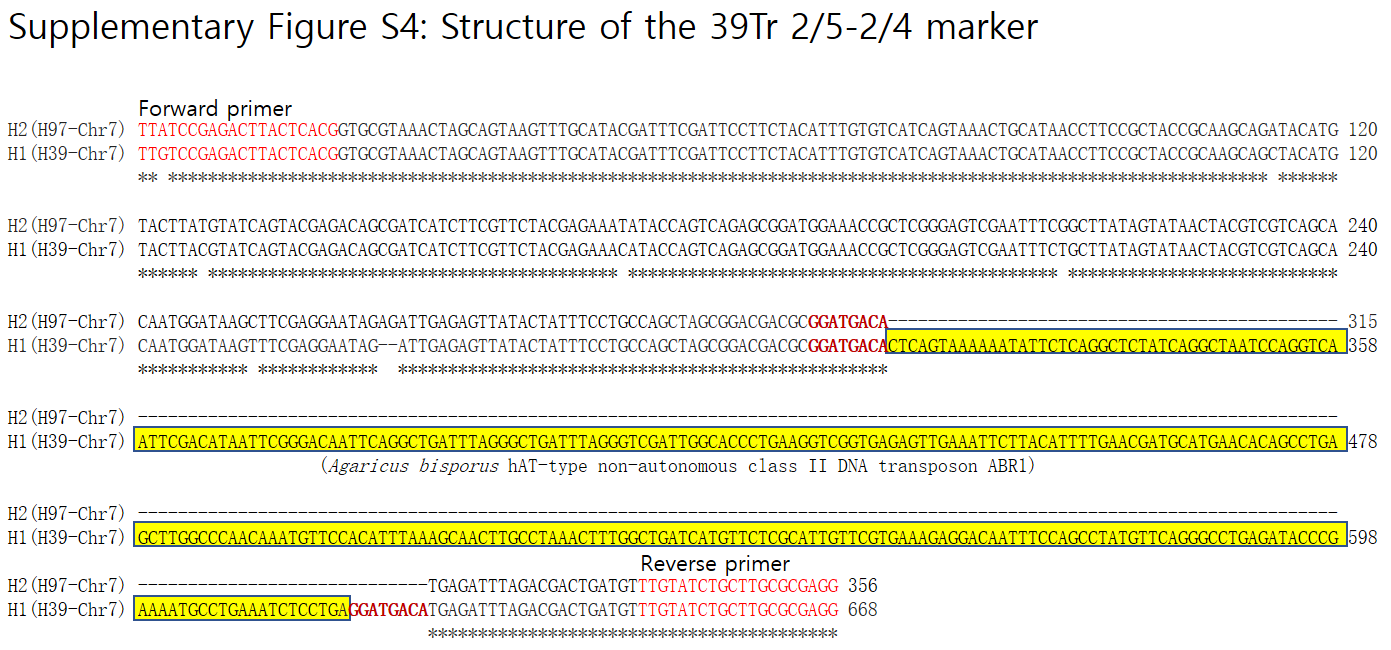

Supplement: Supplementary file 1 [file jof-09-00284-s001.zip › Supplementary Figure S4.TIF]
